# Seroprevalences of specific antibodies against avian pathogens in free-ranging ring-necked pheasants (*Phasianus colchicus*) in Northwestern Germany

**DOI:** 10.1371/journal.pone.0255434

**Published:** 2021-08-04

**Authors:** Friederike Gethöffer, Nele Curland, Ulrich Voigt, Benno Woelfing, Tobias Ludwig, Ursula Heffels-Redmann, Hafez Mohamed Hafez, Michael Lierz, Ursula Siebert

**Affiliations:** 1 Institute for Terrestrial and Aquatic Wildlife Research, University of Veterinary Medicine Hannover, Hannover, Germany; 2 Clinic for Birds, Reptiles, Amphibians and Fish, Justus Liebig Universität Giessen, Giessen, Germany; 3 Institute of Poultry Diseases, Freie Universität Berlin, Berlin, Germany; University of Nicolaus Copernicus in Torun, POLAND

## Abstract

Infectious diseases in captive pheasants (*Phasianus colchicus*) are well known, but there is a lack of knowledge about occurrence and distribution of pathogens in free-ranging pheasants in Germany. We investigated 604 sera from hunted pheasants and 152 sera from wild caught pheasants between 2011 to 2015, with the aim to determine the prevalence of specific antibodies against different viruses: Avian influenza virus (AIV) of subtypes H5, H7, H9, paramyxovirus type 1 (PMV-1), avian encephalomyelitis virus (AEV), infectious bursitis disease virus (IBDV), infectious bronchitis virus (IBV), infectious laryngotracheitis virus (ILTV), avian metapneumovirus (aMPV) and *Salmonella* sp., *Mycoplasma synoviae* (MS) and *Mycoplasma gallisepticum* (MG). In addition, 178 caeca were investigated for *Histomonas meleagridis*. The study reveals an ongoing circulation of IBV in the wild pheasant population during the study. Also high seroprevalences of specific antibodies against aMPV depending on the area and a strong increase in prevalence of IBDV antibodies in sera of pheasants in Lower Saxony were detected. ILTV antibody prevalences differed between areas and AEV antibody detection differed between years significantly, whereas specific antibodies against PMV-1 could not be detected and antibodies against AIV-H5, -H7 and -H9 and *Mycoplasma spp*. were detected in very few cases.

## Introduction

A population decline of free-ranging ring-necked pheasants (*Phasianus colchicus*) can be observed in Northwestern Germany during the last decade [1]. One of the factors potentially influencing the health of free-ranging pheasants and playing a role in the population decline might be pathogen load [2–4].

Little is known about the actual occurrence and pathogenicity of pathogens among free-ranging populations of pheasants in Germany [5]. Only few studies investigated specific antibody prevalence in free-ranging pheasants [6–10]. The originally non-native species is established in Germany as a breeding bird for at least 200 years [11]. Therefore, the ongoing population decline–indicated by hunting index and estimated population density [12]–encouraged us to assess the prevalence of specific antibodies against infectious diseases in the free-ranging pheasant population in Northwestern Germany. Here, a sporadic release of bred pheasants into the wild occurs, but not in an extent comparable to e.g. British populations [13]. Investigations started with an analysis of shot and found dead pheasants, including pathomorphological examinations as well as a screening on different pathogens [2]. The findings of this study indicated that aMPV as well as other viral pathogens might circulate in the wild pheasant populations. According to these previous findings, a main consequence caused by these pathogens would be high mortality rates in offspring and juvenile pheasants [2]. Some of these pathogens might be introduced from poultry farms or back yard poultry [14] by contact, air, dust or dung. In general, transmission of viral infections between domestic and game birds can be a result of direct or indirect contact [5]. The main aims of the present serological survey were to investigate the circulation of different pathogens in free-ranging pheasants and the feasible development of seroprevalences of antibodies against these pathogens during the years of population decline.

Pheasants infected with highly pathogenic avian influenza virus (HPAIV) show severe morbidity and mortality [15]. Newcastle Disease (PMV-1) causes high mortality rates in pheasants [16], too. Depending on the age of infected birds, in pheasant chicks AEV leads to neurological signs, encephalomyelitis, and high mortality [17]. The consequences of an infection with very virulent IBDV for pheasants are not clear, and reaches from a mortality rate of 20% to no clinical signs [8, 18–20]. Coronavirus infections (IBV and Pheasant coronavirus (PhCoV)) cause alterations in the respiratory tract and kidneys [21–24], accompanied with high mortality rates [21, 23] and may affect the egg production [24]. Tracheitis is the main lesion in ILTV-infection and clinical signs vary from mild respiratory signs to severe asphyxia and deaths [25]. The importance of an aMPV infection in respiratory diseases of pheasants is not clarified yet, because it is often squired by other infectious agents such as IBV or bacterial pathogens [26]. Depending on the age of infected bird and on the serovar involved, *Salmonella* spp. induce different mortality rates in pheasants, for example *Salmonella enterica* ssp. *enterica* serovar Agona [27]. The role of *Mycoplasma synoviae* (MS) in pheasants is still unclear, but in red-legged partridges, MS could be isolated related to respiratory diseases [28]. Infection of pheasants with *Mycoplasma gallisepticum* (MG) are described as severe respiratory diseases with high mortality rates [29–32]. Finally, ring-necked pheasants can be infected with *Histomonas meleagridis*. Dead birds showed caecal and liver lesions, but mortality from the disease was not observed [33].

The present study was conducted to obtain more information on the spread of above mentioned pathogens in free-ranging pheasants in Northwestern Germany, and their potential involvement in the current population decline, especially of offspring [2, 34]. This is a first screening on the seroprevalence for different pathogens in adult birds.

## Material and methods

### Sampling and study area

**Fig 1:**
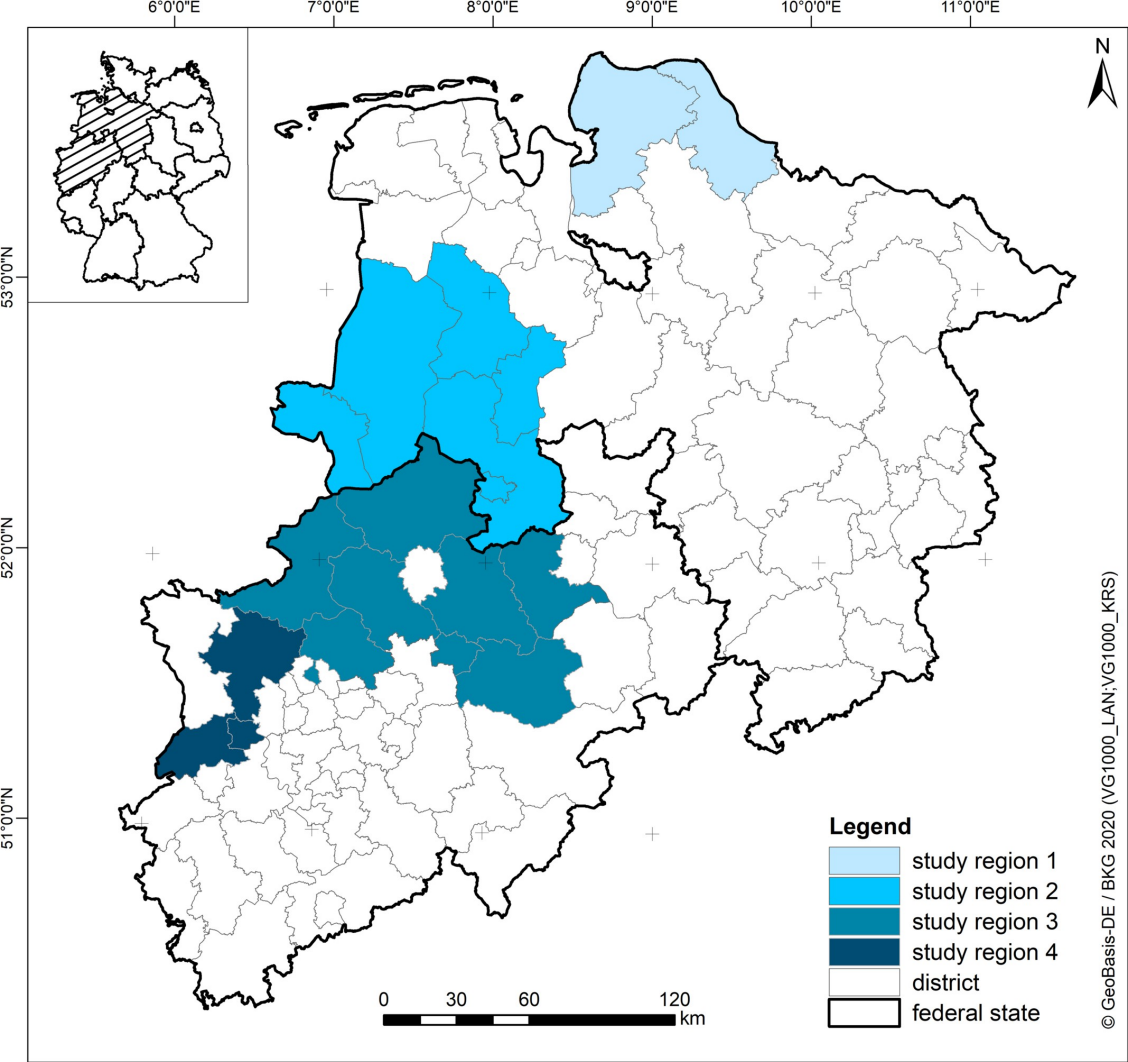
Sampling regions in Northwestern Germany. Situated in the federal states of Lower Saxony and North Rhine-Westphalia.

A total of 604 predominantly male pheasants were sampled from hunting bags in autumn 2011 to 2015 in four different regions in northwest Germany (Fig 1). The hunted pheasants were investigated on the day they were shot. Blood samples were taken from veins and body cavities. Additionally, 152 female pheasants were caught in spring in region 2 in Lower Saxony (Fig 1) under the permit of LAVES 33.12 42502-04-12/0695. Blood samples were taken from Vena cutenea ulnaris superficialis and afterwards the birds were released into the wild again. Blood samples were centrifuged and the sera were stored at -20°C for further investigation.

### Serology

Blood samples were collected from hunted pheasants in autumn and from caught pheasants in spring and examined for antibodies against typical poultry pathogens: Avian influenza virus (AIV) subtypes H5, H7, H9, paramyxovirus type 1 (PMV-1), avian encephalomyelitis virus (AEV), infectious bursitis disease virus (IBDV), infectious bronchitis virus (IBV), infectious laryngotracheitis virus (ILTV), avian metapneumovirus (aMPV) and *Salmonella* sp., *Mycoplasma synoviae* (MS) and *Mycoplasma gallisepticum* (MG). Additionally, caeca were taken from hunted pheasants and checked for *Histomonas meleagridis* by polymerase chain reaction (pcr).

In case of AIV-H5, AIV-H7, AIV-H9, PMV-1 and IBV the hemaggultination inhibition test (HI test) was used according to the methods described by the OIE (2010), while antibodies against AEV and IBDV were detected by using the enzyme linked immunosorbent assay (ELISA, IDEXX) and against aMPV and ILTV by the virus neutralization test (VNT). The rapide plate agglutination test (RPI) was performed for the detection of antibodies against MS, MG and *S*. *pullorum* by the use of antigen from Soleil, France.

### Detection of *H*. *meleagridis* DNA

178 caecum samples were investigated using nested PCR for detection of *H*. *meleagridis* DNA as described [35].

### Statistical analysis

Statistical analyses were conducted in R version 3.5.0 (R Foundation for Statistical Computing) using packages “mgcv” and “multcomp” [36, 37]. We tested if region and year of sampling affect antibody prevalence in male pheasants hunted in autumn using logistic regression models. Separate regression models were set up for each of the five diseases studied (aMPV, AEV, IBDV, IBV and ILTV). Since data was only available for five years (2011–2015), year was coded as a factor variable. Predictor variables in the full model included year, region and their interaction. Model selection was based on the Akaike Information Criterion (AIC) as model selection criterion and an exhaustive screening of all candidate models up to the full model. Reported p-values are based on analysis of deviance. To analyze between which regions or years antibody prevalences differed significantly, a posthoc pairwise comparison with Bonferroni-Holm correction was carried out. Pairwise comparisons were restricted to pairs in which one of the two explanatory variables matches (i.e. pairs that differed in region as well as year were not tested). For female pheasants in spring, results are presented in a descriptive pattern, since the number of samples do not support a strong statistical analysis.

To test for associations between industrial poultry farming and seropositive pheasants, we extended the best logistic regression models (with area and/or region as explanatory variables) for each virus infection type with either farm density or the logarithm of animal density as explanatory variables. The scales considered were districts (DIS) and municipalities (MUN). Beside farm density (F) and the logarithm of animal density (A) at both scales, we distinguished between total hen densities (H) and densities of young hens (YH), laying hens (LH), and broilers (BRO) at the municipality scale (Table 1). We added interaction terms to test for differences between regions from which the samples were collected.

**Table 1:** Comparison of all candidate models for each of the five antibody prevalences.

Response variable	Model	DF	AIC
**IBV**	**year + region**	8	635.1
**IBV**	year x region	12	635.5
**IBV**	region	4	659.2
**IBV**	intercept	1	677.8
**IBV**	year	5	678.5
**AMPV**	**year x region**	11	472.8
**AMPV**	year + region	7	474.7
**AMPV**	year	4	475.4
**AMPV**	region	4	521.3
**AMPV**	intercept	1	525.1
**ILTV**	**region**	4	133.8
**ILTV**	year + region	7	134.2
**ILTV**	year x region	9	136.4
**ILTV**	year	4	145.7
**ILTV**	intercept	1	152.5
**IBDV**	**year x region**	8	303.3
**IBDV**	year + region	6	304.7
**IBDV**	year	3	309
**IBDV**	intercept	1	344.3
**IBDV**	region	4	348.9
**AEV**	**year**	5	330.7
**AEV**	year + region	8	331.9
**AEV**	year x region	12	333.7
**AEV**	region	4	335.2
**AEV**	intercept	1	342.2

DF is the number of degrees of freedom and AIC is the Akaike Information Criterion. For each response variable the selected model is printed in bold.

## Results

From 2011 to 2015, we analyzed 604 sera from hunted pheasants. Additionally, we investigated 152 sera of free-ranging pheasants caught in spring. In total, we tested a maximum of 756 sera for antibodies against up to 11 different pathogens, depending on the recovered volume of blood.

### Sera from hunted pheasants

**Fig 2:**
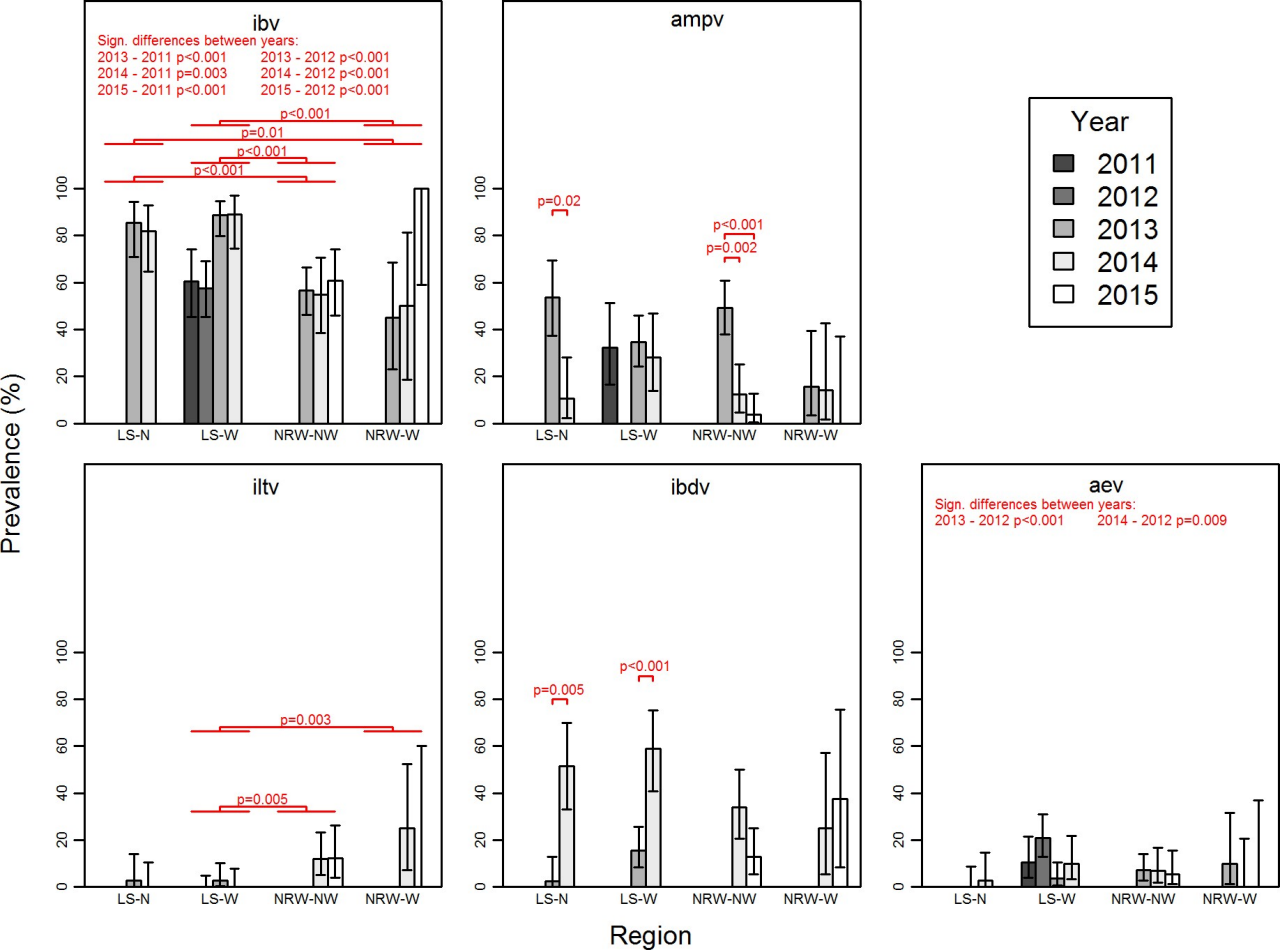
Prevalence of antibodies against the five viral pathogens for different regions and years. Results from the paired post-hoc comparisons with Bonferroni-Holm correction are given in red.

For antibodies to IBV, prevalences ranged from approximately 50% to 100% (Fig 2). The additive model (IBV ~ year + region) fitted the data best (Table 1), indicating that changes in antibody prevalences between years were similar in all regions. Posthoc comparisons showed that antibody prevalences in the years 2013, 2014 and 2015 were significantly higher than in the years 2011 and 2012 (all posthoc p-values were adjusted using the Bonferroni-Holm procedure; Fig 2). Antibody prevalences to IBV were significantly higher in Lower Saxony than in North Rhine-Westphalia (Fig 2).

Observed antibody prevalences to aMPV ranged from 0% to 50% (Fig 2). The model including the interaction between year and region explained antibody prevalences best. In the north of Lower Saxony and in the northwestern part of North Rhine-Westphalia prevalences of antibodies to aMPV decreased sharply from 50% in 2013 to levels below 15% in 2014 and 2015 (Fig 2). No pronounced changes in prevalences were observed in the western parts of Lower Saxony and North Rhine-Westphalia.

Prevalences of antibodies to ILTV differed significantly between regions but not between years (Table 1 and Fig 2). Prevalences were below 5% in Lower Saxony but reached 15% and 30% in the northwestern and western parts of North Rhine-Westphalia (Fig 2).

For antibodies to IBDV the model including the interaction between year and region was selected (Table 1). In Lower Saxony a significant increase in prevalences was observed from prevalences below 18% in 2013 to levels above 50% in 2014 (Fig 2). No such increase in antibody prevalences was observed in North Rhine-Westphalia.

Prevalences of antibodies to AEV differed significantly between years but not between regions (Table 1). The highest prevalence of 22% was observed in 2012 (Fig 2). Prevalences in other years were significantly lower (Fig 2).

There were no findings of antibodies against PMV1 or AIV H7 and H9. The prevalence of antibodies against AIV H5 among hunted pheasants was 0.5%.

Antibodies against *Mycoplasma synoviae* (MS) were detected in three of the game birds of season 2011, while no antibodies against *Mycoplamsa gallisepticum* (MG) were found.

In three cases (n = 178, 1.7%) *Histomonas meleagridis* DNA was detected in nested PCR. Positive samples originated from region 3 in 2011 from hunted pheasants.

To find out if there would be a statistical link between our findings in antibody detection and the occurrence of poultry in the different regions, we checked data on regional farming composition of Lower Saxony and North Rhine—Westphalia [38]:

Data on poultry comprised numbers of animals as well as facilities and could be subsided into chicken (containing young hens, laying hens and broilers) and turkey. We found weak positive relationships only between broiler farm density and AMPV detection in region 2 (Table 2). Additionally, we found negative relationships between the number of seropositive samples and poultry farming indicator variables. For example, the probability of IBV, ILTV, IBDV and AMPV—seropositivity decreased with increasing total, laying hen or broiler farm or even animal density at district or municipality level (Table 2, S1 Tables and S1 Figs in S1 File). For the number of AEV positive serum samples and poultry farming indicators, no effect could be detected.

**Table 2:** Variables of farm and poultry density used for modeling the seropositivity in pheasants.

covariate	explanation	n	mean	sd	median	min	max
DIS_F_H	total farms/ 100 km² district	604	15.36	4.35	15.00	4.00	23.00
DIS_A_H	log10(hens)/ km² district	568	3.37	0.52	3.15	2.27	4.1
MUN_F_H	total farms/ 10 km² municipality	604	1.39	0.68	1.33	0.32	4.54
MUN_F_YH	young hen farms/ 10 km² municip.	604	0.16	0.27	0.06	0.00	1.54
MUN_F_LH	laying hen farms/ 10 km² municip.	604	0.90	0.53	0.84	0.08	3.36
MUN_F_BRO	broiler farms/ 10 km² municip.	604	0.42	0.43	0.28	0.00	1.53
MUN_A_H	log10(hens)/ km² municipality	488	3.27	1.03	3.65	0.00	4.35
MUN_A_YH	log10(young hen)/ km² municipality	15	3.42	0.04	3.45	3.37	3.45
MUN_A_LH	log10(laying hen)/ km² municipality	154	2.53	1.28	2.78	0.00	3.86
MUN_A_BRO	log10(broiler)/ km² municipality	112	3.50	0.61	3.57	2.34	4.21

### Sera from trapped pheasants

From 2011 to 2015, two female pheasants in 2011 and four female pheasants in 2013 were tested seropositive for IBV antibodies, revealing a percentage of 7% and 12% positive individuals per season. For aMPV, IBDV and AEV antibodies, the results do show a range between 3 and 23%. ILTV antibodies were only found in spring 2015, reaching 27% of the tested animals. No AIV or PMV1—antibodies were detected. Antibodies against *Mycoplasma synoviae* (MS) were detected in 6 female pheasants in spring during several years (Table 3). Antibodies against *Mycoplasma gallisepticum* (MG) were detected in 5 out of 136 sera of caught pheasants (3.7%) (Table 4).

**Table 3:** Number and percentage of serum samples tested positive for antibodies against IBV, aMPV, ILTV, IBDV and AEV during spring sampling. Only trapped pheasants were included.

year	n	IBV		aMPV		ILTV		IBDV		AEV	
		pos	%	pos	%	pos	%	pos	%	pos	%
**2011**	28	2	**7.1**	5	**17.9**	0	**0**	3	**10.7**	2	**7.1**
**2012**	28	0	**0**	0	**0**	0	**0**	0	**0**	2	**7.1**
**2013**	33	4	**12.1**	4	**12.1**	0	**0**	2	**6.1**	1	**3.0**
**2014**	30	0	**0**	7	**23.3**	0	**0**	3	**10.0**	2	**6.7**
**2015**	33	0	**0**	5	**15.2**	9	**27.3**	3	**9.1**	0	**0**
**sum**	152	6	**4**	21	**14**	9	**6**	11	**7**	7	**5**

**Table 4:** Number of serum samples tested positive for antibodies against *Mycoplasma synoviae* (MS) and *Mycoplasma gallisepticum* (MG) during spring sampling. Only trapped pheasants were included.

year	n	pos MS	% MS	n	pos MG	% MG
**2011**	20	2	**10**	21	2	**0.1**
**2012**	26	0	**0**	26	0	**0**
**2013**	27	3	**11.1**	28	2	**0.1**
**2014**	29	1	**3.4**	28	1	**0**
**2015**	31	0	**0**	33	0	**0**
**sum**	**133**	**6**	**5**	**136**	**5**	**4**

## Discussion

The serological survey of free-ranging pheasants in Northwestern Germany provides evidence of antibodies against different respiratory diseases (IBV, aMPV, ILTV) as well as antibodies against IBDV, AEV and *Mycoplasma* (MS, MG). In addition, some positive evidences of *Histomonas meleagridis* DNA could be detected from caecal contents of tested samples.

A main result of the survey is the ongoing increase of seroconverted free-ranging pheasants for IBV during the investigated years. The results especially show a high percentage of antibodies against IBV in the male pheasants hunted in autumn, while in spring only few pheasants were tested positive for antibodies. In this group of pheasants caught and investigated in spring, we see a small increase in distribution of antibodies against IBV from 2011 to 2013, but in the following two years, all of the investigated samples were negative.

Spring and summer represent the main seasons for new infections. During this time, offspring is raised, which is highly susceptible for IBV. Apart from chickens, pheasants are the only avian species that is considered as a second natural host for IBV [39]. As a result, an infection could lead to high mortality rates especially in young pheasants and, furthermore, weaken the adult birds becoming an easy prey during and after the time of breeding. Subsequently, too little offspring could be raised to preserve the population [2].

Regarding the difference of results between pheasants investigated in early spring and in autumn, e.g. an infection might have occurred during spring and summer, or vaccinated pheasants might have been released so that birds with antibodies arised from vaccination were investigated. Winter mortality is high in released pheasants [40], in the UK, 42% up to 61% of released pheasants died until February [41]. This might lead to a distinction of released pheasants during the spring sampling and therefore explain the different findings in spring and autumn. Based on investigations on antibodies it is not possible to differentiate between antibody reactions against wild-type virus or vaccine strains or to determine the pathogenicity for pheasants. So far, the negative relationship of IBV seropositivity and increasing poultry densities seems contrary to expectations at a first sight. Going more into details, this relation might be especially true for young and laying hens, as this would be an explanation because of the thorough vaccination program of this farm birds. Nevertheless, our sampling design was not designated to answer this question in beforehand and further studies on this would be helpful.

Antibodies against infectious bursal disease virus (IBDV) were detected in 25.8% of investigated hunted pheasants and in 7.8% of pheasants in spring. A small study in two sampling areas in China showed antibodies against IBDV in 35% of tested samples (n = 40) from free-ranging pheasants [8]. In Ireland, antibodies against IBDV were found in 80% of investigated wild pheasants [42]. Again, the consequences of an infection with IBDV for pheasants are not clear, literature reaches from mortality rate of 20% [8] to no clinical signs [18, 19]. Again, number of seropositive samples are only weakly connected with poultry keeping, in this case of laying hens. This might be a hint on either earlier or no exchange, since only young hens are vaccinated against IBDV generally.

Regarding the effects of IBDV in chickens, especially the immunosuppression is affecting the population, and first of all young chicks are concerned [19]. Assuming similar consequences in pheasants, there would be less protection from maternal antibodies, and thereby vulnerable offspring. Considering the high number of different pathogens circulating in the pheasant population, a weakened immune system in offspring could have fatal consequences for a population recruitment.

Antibodies against avian metapneumovirus (aMPV) were detected in 28.6% of hunted pheasants and in 13.7% pheasants in spring. A survey of antibodies against avian pneumovirus in free-ranging pheasants in the Po Valley in Italy showed a prevalence of 1.1% in 1992 and 0.8% in 1994 [6]. In pheasants released from farms, prevalence was much higher which according to the authors might indicate that an infection in the wild comes from game farms [6]. In our study, there is evidence of significant higher seropositive sample sizes and high density of mast chicken as well as turkey farms in region 2, being in line with susceptibility of these animals for the disease, therefore an exchange of some kind cannot be excluded. This virus is usually found in co-infections with other viruses like IBV or bacterial infection in the respiratory tract of pheasants. As it is not common in Germany to vaccinate on poultry farms against aMPV, it seems not likely to be a vaccine-derived strain in this case.

To our knowledge, there are no comparable studies of seroprevalence of antibodies against ILTV and AEV in free-ranging pheasants available so far. Depending on age of infection, in pheasant chicks AEV leads to neurological signs, encephalomyelitis accompanied with partly, and/or high mortality. Neurological signs include tremor, incoordination and falling on their back unable to stand up [17]. Offspring with this infection has no chance to survive in the wild. In chickens and presumably also in pheasants, there is an age dependent resistance against clinical signs caused by AEV infection. In commercial poultry, vaccination is common using a full pathogenic strain in chicks around 10 weeks of age [17]. As AEV can be vertically transmitted via hatching eggs, poultry dung brought on the fields for fertilization can spread the vaccination-strain to free-ranging pheasants causing seroconversion in older animals and clinical signs in susceptible young chicks. We found no significant statistical interaction of numbers of seropositive samples and densities of young chicken or mast chicken farms, although it has to be kept in mind that the study design was not laid up to this aspect.

Clinical signs of ILTV infection vary from mild respiratory signs to severe asphyxiation and death [25]. This is one further pathogen which can weaken the population and for which antibodies have been detected in this study.

For MG-antibodies, none of the investigated hunted pheasants was positive, whereas 3.7% of investigated pheasants in spring (n = 136) showed a positive result. An Italian study came to a similar result, the pheasant tested positive had been reared and released in the wild [9]. We cannot exclude that either the five positive female pheasants in our study were equally reared and released into the wild already carrying these antibodies, nor state if they were infected naturally in the wild. But a spread of this pathogen among the wild pheasant population would lead to severe respiratory diseases and high mortality rates among the wild population.

The prevalence of antibodies against *Mycoplasma synoviae* (MS) is rather low in our investigations. From red-legged partridges suffering from respiratory distress, MS could be isolated [28]. Even if the role of MS in pheasants is still unclear, an effect on pheasants health has to be considered. But regarding the low percentage of positive samples, in the actual decline MS seems to be non-relevant. In addition, the first study on diseases in free living ring necked pheasants did not discover MG nor MS in any of the birds sampled [2], so the occurrence of MG or MS in the wild pheasant population is highly unlikely.

Antibodies against Newcastle Disease (PMV-1) were not detected in our investigation. So currently, this seems to play not an important part concerning the population decline during the years. Since there is no information so far if released pheasants are vaccinated against Newcastle Disease, we can only guess that either they are not since if there is a vaccination done, there should be a detection of antibodies in the wild pheasants population.

Avian influenza antibodies against subtype AIV H5 were detected in a few pheasants (0.5%). On the other hand, no antibodies against subtypes H7 and H9 were detected. In a study in Italy, no sex-related difference was found, whereas a higher susceptibility of juveniles was suggested [10]. Since sporadic detection of H5 subtypes in wild birds seems to be common [43–45], AIV does not seem to be of major influence on the population decline.

In three cases (n = 178, 1.7%) of tested caeca, *Histomonas meleagridis* DNA was detected. *H*. *meleagridis*, causing “blackhead-disease” in turkey [46] although mortality was not observed in pheasants [33], representing an important host and contaminator through *Heterakis* eggs [47, 48]. The very low occurrence of this pathogen should not result in higher mortality or even population decline in free living pheasants.

## Conclusions

Considering the occurrence and dissemination of the mentioned diseases over the whole study area, an impact on population development of pheasants seems likely. The results presented in this study may reflect the serological response after natural infection, after possible vaccination (in a limited number of cases) or due to the possible spread of live vaccine strains (e.g. IBV, aMPV, ILT), as live vaccines are commonly used in poultry industry. Nevertheless, the presented prevalence and number of various pathogens suggest that the population is weakened by diseases. Some of the diseases especially affect the offspring during their first weeks of life. Additionally, free-ranging pheasants deal with different influences and stresses of the environment. Strong winters and wet springs reduce the winter survival of adult birds and the survival of offspring during the first weeks of life [49–51]. Furthermore, predation has a strong influence, especially during breeding time for hens sitting on the ground, and for offspring, unable to fly [34, 52, 53]. On top, landscape and agriculture have changed during the last years, so less protective cover and structure of the landscape reduces possibilities to hide and find protection. Additionally, less variability in crops in combination with intensified pesticide use reduce the food variability of insects and make it harder for pheasants to find adequate nutriment [54, 55]. The latter is especially true for chicks which depend on insect protein during their first week of life and lacking of this source may negatively influence the immune system. The combination of the mentioned factors might influence the strength and resistance of free-ranging pheasants and the detected increase of seroconversion of specific antibodies in this study shows the spread of pathogens over the study area. According to many different studies, the enhanced susceptibility of birds to infectious diseases is an effect of various immunosuppressors [56–58]. In conclusion, we consider the detected antibodies against important infectious diseases in non- vaccinated free-ranging pheasants to be a further cofactor of a multivariate process in population decline.

## Supporting information

S1 File Possible influence of poultry farming on seropositivity. (DOCX)
