# Supplementary material for: Seroprevalences of specific antibodies against avian pathogens in free-ranging ring-necked pheasants (Phasianus colchicus) in Northwestern Germany
Source: PLoS One. 2021 Aug 4;16(8):e0255434. doi: 10.1371/journal.pone.0255434 (PMC8336876; doi:10.1371/journal.pone.0255434)
Supplement: S1 File — (DOCX) [file pone.0255434.s001.docx]

## **S1 File. Possible influence of poultry farming on seropositivity**

To test for associations between industrial poultry farming and seropositive pheasants, we extended the best logistic regression models (with area and region as explanatory variables) for each virus infection type with either farm density or the logarithm of animal density as explanatory variables. The scales considered were districts (DIS) and municipalities (MUN). Beside farm density (F) and the logarithm of animal density (A) at both scales, we distinguished between total hen densities (H) and densities of young hens (YH), laying hens (LH), and broilers (BRO) at the municipality scale (Table S1.1). We added interaction terms to test for differences between regions from which the samples were collected.

**S1 Table 1. Variables of farm and animal density used for modeling the seropositivity in pheasants, together with their sample sizes (n) and location parameters.**

| covariate | explanation | n | mean | sd | median | min | max |
| --- | --- | --- | --- | --- | --- | --- | --- |
| DIS_F_H | total farms/ 100 km² district | 604 | 15.36 | 4.35 | 15.00 | 4.00 | 23.00 |
| DIS_A_H | log10(hens)/ km² district | 568 | 3.37 | 0.52 | 3.15 | 2.27 | 4.1 |
| MUN_F_H | total farms/ 10 km² municipality | 604 | 1.39 | 0.68 | 1.33 | 0.32 | 4.54 |
| MUN_F_YH | young hen farms/ 10 km² municip. | 604 | 0.16 | 0.27 | 0.06 | 0.00 | 1.54 |
| MUN_F_LH | laying hen farms/ 10 km² municip. | 604 | 0.90 | 0.53 | 0.84 | 0.08 | 3.36 |
| MUN_F_BRO | broiler farms/ 10 km² municip. | 604 | 0.42 | 0.43 | 0.28 | 0.00 | 1.53 |
| MUN_A_H | log10(hens)/ km² municipality | 488 | 3.27 | 1.03 | 3.65 | 0.00 | 4.35 |
| MUN_A_YH | log10(young hen)/ km² municipality | 15 | 3.42 | 0.04 | 3.45 | 3.37 | 3.45 |
| MUN_A_LH | log10(laying hen)/ km² municipality | 154 | 2.53 | 1.28 | 2.78 | 0.00 | 3.86 |
| MUN_A_BRO | log10(broiler)/ km² municipality | 112 | 3.50 | 0.61 | 3.57 | 2.34 | 4.21 |

We found weak positive relationships only between broiler farm density and AMPV detection in region 2 (S1 Fig 4). Additionally, we found negative relationships between seropositivity and poultry farming indicator variables. For example, probability of IBV and IBDV decreased with increasing laying hen farm density at the municipality level (S1 Fig 1 and S1 Fig 9). IBV seropositivity also decreased with increasing broiler farm density at the municipality level (S1 Fig 2). Total hen density at the district level was a negative predictor of AMPV (S1 Fig 3), ILTV (S1 Fig 5), and IBDV in region 2 (S1 Fig 7). Only in region 1 there was a tendency for increasing IBDV probability with that variable. Finally, IBDV was also negatively related to total hen farm density both, at the district (S1 Fig 6) and municipality level (S1 Fig 8).

# **IBV**

## Best model as starting point:

IBV ~ year + region; AIC = 632.87

**S1 Table 2. Model for farm density of laying hens at municipality level** (MUN_F_LH) Null deviance: 671.99 on 536 degrees of freedom, Residual deviance: 605.08 on 525 degrees of freedom, AIC: 629.08.

| Parameter | Estimate | Std.Error | z value | Pr(>\|z\|) | sign. Value |
| --- | --- | --- | --- | --- | --- |
| (Intercept) | -0.6731 | 0.7172 | -0.938 | 0.347993 |  |
| year_factor2012 | -0.1059 | 0.3903 | -0.271 | 0.786024 |  |
| year_factor2013 | 16.515 | 0.4343 | 3.803 | 0.000143 | *** |
| year_factor2014 | 15.864 | 0.4668 | 3.398 | 0.000678 | *** |
| year_factor2015 | 20.975 | 0.5306 | 3.953 | 7.72e-05 | *** |
| region_2 | 22.507 | 0.7650 | 2.942 | 0.003258 | ** |
| region_3 | -0.8693 | 0.6883 | -1.263 | 0.206627 |  |
| region_4 | -0.6713 | 0.8584 | -0.782 | 0.434197 |  |
| MUN_F_LH | 0.7871 | 0.5984 | 1.315 | 0.188399 |  |
| region_2:MUN_F_LH | -22.792 | 0.7722 | -2.952 | 0.003161 | ** |
| region_3:MUN_F_LH | -0.7140 | 0.6591 | -1.083 | 0.278665 |  |
| region_4:MUN_F_LH | -0.9909 | 12.602 | -0.786 | 0.431690 |  |


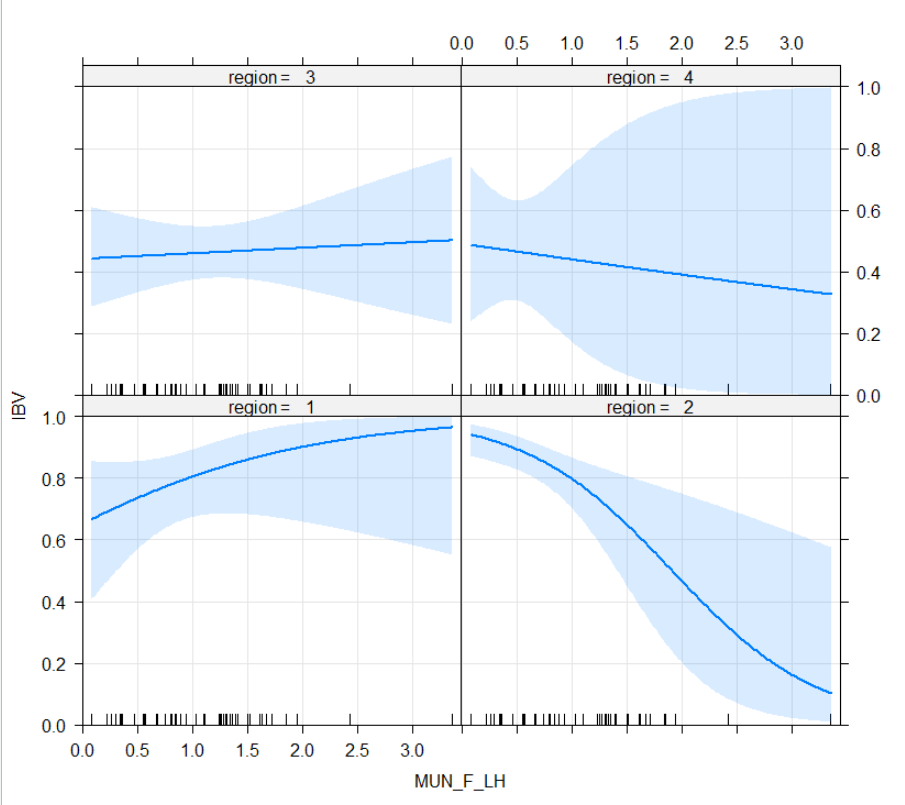


**S1 Fig 1. Significantly decreasing probability for IBV-seropositivity in region 2 with increasing laying hen - farm density at the municipality level.** The increase in region 1 is not significant (p=0.195).

**S1 Table 3. Model for farm density of broilers at municipality level** (MUN_F_BRO). Null deviance: 671.99 on 536 degrees of freedom, Residual deviance: 612.28 on 528 degrees of freedom, AIC: 630.28.

| Parameter | Estimate | Std.Error | z value | Pr(>\|z\|) | sign. Value |
| --- | --- | --- | --- | --- | --- |
| (Intercept) | 0.1683 | 0.5330 | 0.316 | 0.752120 |  |
| year_factor2012 | -0.1265 | 0.3853 | -0.328 | 0.742635 |  |
| year_factor2013 | 16.080 | 0.4306 | 3.734 | 0.000189 | *** |
| year_factor2014 | 15.754 | 0.4577 | 3.442 | 0.000578 | *** |
| year_factor2015 | 20.654 | 0.5273 | 3.917 | 8.98e-05 | *** |
| region_2 | 0.8019 | 0.4697 | 1.707 | 0.087770 | . |
| region_3 | -14.870 | 0.3604 | -4.126 | 3.70e-05 | *** |
| region_4 | -14.946 | 0.4644 | -3.218 | 0.001289 | ** |
| MUN_F_BRO | -0.6904 | 0.3209 | -2.152 | 0.031435 | * |


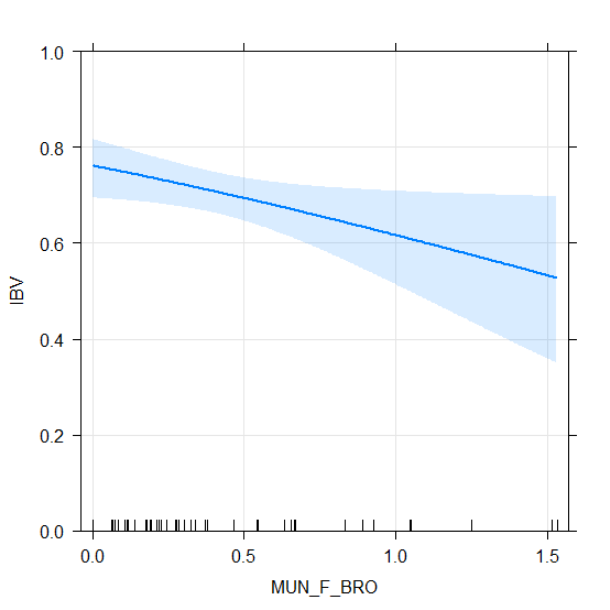


**S1 Fig 2. The probability for IBV-seropositivity decreases with increasing broiler farm density at the municipality level.**

# **AMPV**

## Best models as starting point:

AMPV ~ year + region; AIC = 471.81

**S1 Table 4. Model for animal density of total hens at district level** (DIS_A_H). Null deviance: 491.18 on 405 degrees of freedom, Residual deviance: 425.89 on 398 degrees of freedom (29 observations deleted due to missingness), AIC: 441.89

| Parameter | Estimate | Std.Error | z value | Pr(>\|z\|) | sign. Value |
| --- | --- | --- | --- | --- | --- |
| (Intercept) | 610.568 | 246.305 | 2.479 | 0.013178 | * |
| year_factor2013 | 0.34905 | 0.44939 | 0.777 | 0.437316 |  |
| year_factor2014 | -0.88301 | 0.50564 | -1.746 | 0.080755 | . |
| year_factor2015 | -287.724 | 0.86854 | -3.313 | 0.000924 | *** |
| region_2 | 163.345 | 0.79439 | 2.056 | 0.039760 | * |
| region_3 | -0.06209 | 0.33201 | -0.187 | 0.851653 |  |
| region_4 | -251.912 | 0.84764 | -2.972 | 0.002960 | ** |
| DIS_A_H | -219.465 | 0.81120 | -2.705 | 0.006821 | ** |


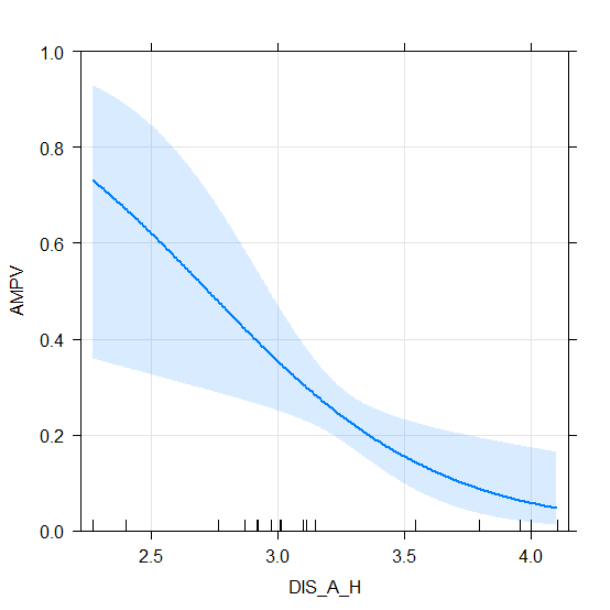


**S1 Fig 3. The probability for AMPV-seropositivity decreases with increasing hen density at the district level.**

**S1 Table 5. Model for farm density of broilers at municipality level (**MUN_F_BRO) Null deviance: 519.97 on 434 degrees of freedom, Residual deviance: 448.99 on 424 degrees of freedom, AIC: 470.99.

| Parameter | Estimate | Std.Error | z value | Pr(>\|z\|) | sign. Value |
| --- | --- | --- | --- | --- | --- |
| (Intercept) | -0.3005 | 0.5376 | -0.559 | 0.57624 |  |
| year_factor2013 | 0.4052 | 0.4512 | 0.898 | 0.36911 |  |
| year_factor2014 | -10.519 | 0.5139 | -2.047 | 0.04069 | * |
| year_factor2015 | -27.770 | 0.8677 | -3.200 | 0.00137 | ** |
| region_2 | -11.618 | 0.4826 | -2.408 | 0.01606 | * |
| region_3 | -0.1755 | 0.4254 | -0.413 | 0.67991 |  |
| region_4 | -34.244 | 14.575 | -2.349 | 0.01880 | * |
| MUN_F_BRO | -0.8820 | 0.7570 | -1.165 | 0.24400 |  |
| region_2:MUN_F_BRO | 18.523 | 0.8857 | 2.091 | 0.03650 | * |
| region_3:MUN_F_BRO | 0.4960 | 17.769 | 0.279 | 0.78015 |  |
| region_4:MUN_F_BRO | 166.032 | 93.355 | 1.779 | 0.07532 | . |


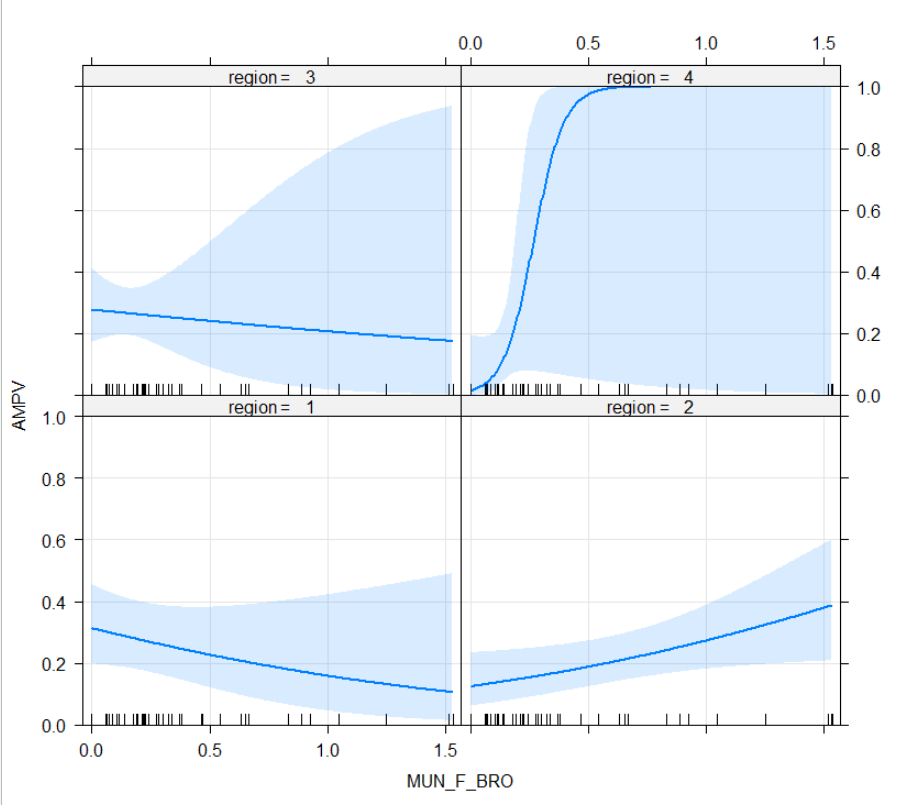


**S1 Fig 4. Slightly increasing probability for AMPV-seropositivity in region 2 with increasing broiler - farm density at the municipality level.**

# **ILTV**

## Best model as starting point:

ILTV ~ region; AIC = 133.6920

**S1 Table 6: Model for animal density of total hens at district level** (DIS_A_H). Null deviance: 136.30 on 352 degrees of freedom, Residual deviance: 102.47 on 348 degrees of freedom (20 observations deleted due to missingness), AIC: 112.47.

| Parameter | Estimate | Std.Error | z value | Pr(>\|z\|) | sign. Value |
| --- | --- | --- | --- | --- | --- |
| (Intercept) | 165.016 | 78.542 | 2.101 | 0.0356 | * |
| region_2 | 52.266 | 22.469 | 2.326 | 0.0200 | * |
| region_3 | 22.093 | 10.689 | 2.067 | 0.0387 | * |
| region_4 | -0.9813 | 19.160 | -0.512 | 0.6085 |  |
| DIS_A_H | -70.531 | 26.843 | -2.627 | 0.0086 | ** |


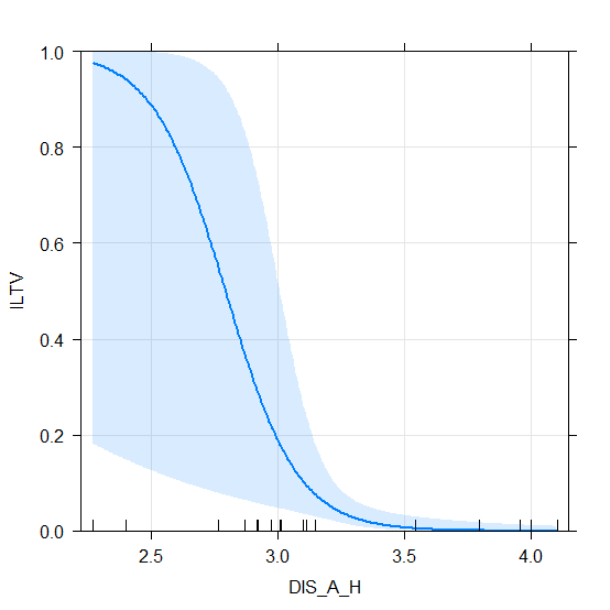


**S1 Fig 5. The probability for ILTV-seropositivity decreases with increasing hen density at the district level.**

# **IBDV**

## Best model as starting point:

IBDV ~ year + region; AIC = 300.29

**S1 Table 7: Model for farm density of total hens at district level** (DIS_F_H). Null deviance: 339.00 on 298 degrees of freedom, Residual deviance: 276.16 on 292 degrees of freedom (510 observations deleted due to missingness),AIC: 290.16.

| Parameter | Estimate | Std.Error | z value | Pr(>\|z\|) | sign. Value |
| --- | --- | --- | --- | --- | --- |
| (Intercept) | -0.83182 | 0.67588 | -1.231 | 0.218425 |  |
| DIS_F_H | -0.17954 | 0.05377 | -3.339 | 0.000841 | *** |
| region_2 | 205.653 | 0.58673 | 3.505 | 0.000456 | *** |
| region_3 | 0.11501 | 0.51176 | 0.225 | 0.822193 |  |
| region_4 | -0.73520 | 0.64434 | -1.141 | 0.253867 |  |
| year_factor2014 | 267.029 | 0.42876 | 6.228 | 4.73e-10 | *** |
| year_factor2015 | 169.649 | 0.63287 | 2.681 | 0.007348 | ** |


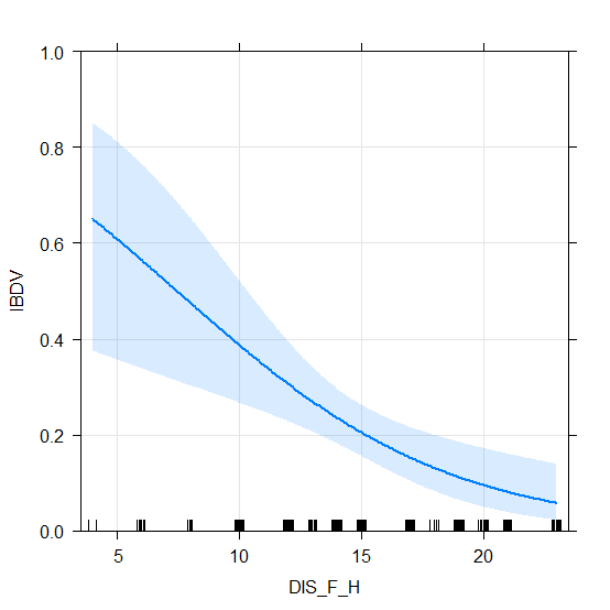


**S1 Fig 6. The probability for IBDV-seropositivity decreases with increasing hen density at the district level.**

**S1 Table 8: Model for animal density of total hens at district level** (DIS_A_H). Null deviance: 317.15 on 287 degrees of freedom, Residual deviance: 257.37 on 278 degrees of freedom (11 observations deleted due to missingness), AIC: 277.37.

##

| Parameter | Estimate | Std.Error | z value | Pr(>\|z\|) | sign. Value |
| --- | --- | --- | --- | --- | --- |
| (Intercept) | -161.744 | 72.033 | -2.245 | 0.02474 | * |
| year_factor2014 | 27.028 | 0.4419 | 6.117 | 9.56e-10 | *** |
| year_factor2015 | 24.003 | 0.7303 | 3.287 | 0.00101 | ** |
| region_2 | 256.494 | 88.241 | 2.907 | 0.00365 | ** |
| region_3 | 32.989 | 133.036 | 0.248 | 0.80416 |  |
| region_4 | 140.192 | 221.307 | 0.633 | 0.52643 |  |
| DIS_A_H | 44.573 | 23.749 | 1.877 | 0.06054 | . |
| region_2:DIS_A_H | -74.317 | 27.285 | -2.724 | 0.00646 | ** |
| region_3:DIS_A_H | -15.015 | 44.042 | -0.341 | 0.73317 |  |
| region_4:DIS_A_H | -50.034 | 92.461 | -0.541 | 0.58841 |  |


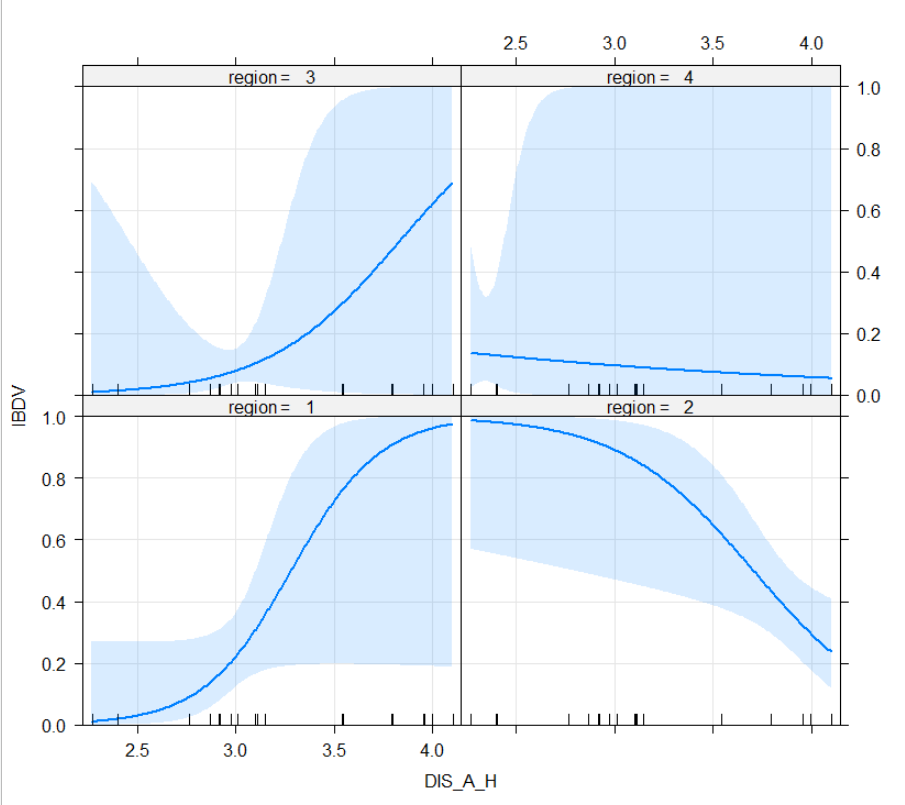


**S1 Fig 7. Decreasing probability for IBDV-seropositivity in region 2 with increasing hen density at the district level.**

**S1 Table 9: Model for farm density of total hens at municipality level** (MUN_F_H). Null deviance 339.00 on 298 degrees of freedom, Residual deviance: 284.61 on 292 degrees of freedom (510 observations deleted due to missingness), AIC: 298.61.

| Parameter | Estimate | Std.Error | z value | Pr(>\|z\|) | sign. Value |
| --- | --- | --- | --- | --- | --- |
| (Intercept) | -22.868 | 0.4907 | -4.661 | 3.15e-06 | *** |
| MUN_F_H | -0.4092 | 0.2268 | -1.804 | 0.0713 | . |
| region_2 | 0.9599 | 0.4444 | 2.160 | 0.0308 | * |
| region_3 | -0.3027 | 0.4903 | -0.617 | 0.5370 |  |
| region_4 | -0.4901 | 0.6180 | -0.793 | 0.4278 |  |
| year_factor2014 | 25.224 | 0.4168 | 6.052 | 1.43e-09 | *** |
| year_factor2015 | 14.780 | 0.6188 | 2.389 | 0.0169 | * |


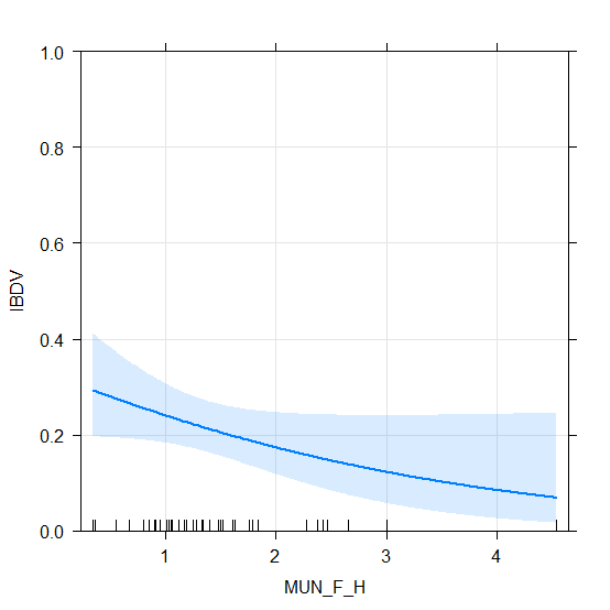


**S1 Fig 8. Decreasing probability for IBDV-seropositivity with increasing hen farm density at the municipality level.**

**S1 Table 10. Model for farm density of laying hens at municipality level** (MUN_F_LH). Null deviance: 339.00 on 298 degrees of freedom, Residual deviance: 280.58 on 292 degrees of freedom (510 observations deleted due to missingness), AIC: 294.58.

##

| Parameter | Estimate | Std.Error | z value | Pr(>\|z\|) | sign. Value |
| --- | --- | --- | --- | --- | --- |
| (Intercept) | -19.534 | 0.5123 | -3.813 | 0.000137 | *** |
| MUN_F_LH | -0.8160 | 0.3160 | -2.582 | 0.009822 | ** |
| region_2 | 0.5850 | 0.4229 | 1.383 | 0.166602 |  |
| region_3 | -0.1829 | 0.4900 | -0.373 | 0.708915 |  |
| region_4 | -0.5993 | 0.6238 | -0.961 | 0.336743 |  |
| year_factor2014 | 25.189 | 0.4170 | 6.040 | 1.54e-09 | *** |
| year_factor2015 | 14.246 | 0.6218 | 2.291 | 0.021966 | * |


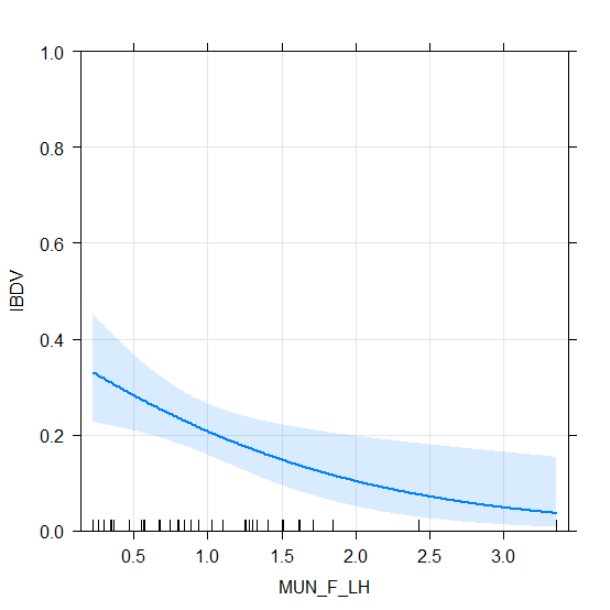


**S1 Fig 9. Decreasing probability for IBDV-seropositivity with increasing laying hen farm density at the municipality level.**
